# "*not one size fits all*” The challenges of measuring paediatric health-related quality of life and the potential role of digital ecological momentary assessment: a qualitative study

**DOI:** 10.1007/s11136-023-03535-6

**Published:** 2023-10-27

**Authors:** Holly Fraser, Lauren Thompson, Esther Crawley, Matthew J. Ridd, Amberly Brigden

**Affiliations:** 1 Digital Health, Faculty of Engineering, School of Computer Science, Electrical and Electronic Engineering, University of Bristol, Amberly Brigden, 1 Cathedral Square, Bristol, BS1 5DD UK; 2 Faculty of Health Sciences, Centre for Child and Adolescent Health, University of Bristol, Bristol, BS8 2PS UK; 3 Faculty of Health Sciences, Centre for Academic Primary Care, University of Bristol, Bristol, BS8 2PS UK

**Keywords:** Health-related quality of life, HRQoL, Paediatrics, Ecological momentary assessment, Digital health, Qualitative

## Abstract

**Purpose:**

To explore the views of clinicians and researchers about the challenges of measuring health-related quality of life (HRQoL) in children (5–11 years) and to explore whether digital ecological momentary assessment (EMA) could enhance HRQoL measurement.

**Methods:**

Semi-structured qualitative interviews with 18 professionals (10 academics/researchers, four clinicians, four with both professional backgrounds) experienced in child HRQoL measurement. We analysed data thematically.

**Results:**

Theme One describes the uncertainty around conceptualising HRQoL for children and which domains to include; the greater immediacy and sensitivity of children’s reflections on their HRQoL, leading to high variability of the construct; and the wide individual differences across childhood, incongruent with fixed HRQoL measures. Theme Two describes the challenges of proxy reporting, questioning whether proxies can meaningfully report a child’s HRQoL and reflecting on discrepancies between child and proxy reporting. Theme Three covers the challenge of interpreting change in HRQoL over time; does a change in HRQoL reflect a change in health, or does this reflect developmental changes in how children report HRQoL. Theme Four discusses digital EMA for HRQoL data capture. In-the-moment, repeated measurement could provide rich data and address challenges of recall, ecological validity and variability; passive data could provide objective markers to supplement subjective responses; and technology could enable personalisation and child-centred design. However, participants also raised methodological, practical and ethical challenges of digital approaches.

**Conclusion:**

Digital EMA may address some of the challenges of HRQoL data collection with children. We conclude by discussing potential future research to explore and develop this approach.

**Supplementary Information:**

The online version contains supplementary material available at 10.1007/s11136-023-03535-6.

## Introduction

Health-related quality of life (HRQoL) is multidimensional, including social, emotional, cognitive, and physical functioning [1]. HRQoL is reported by the individual (or proxy), influenced by their experiences, beliefs, and perceptions [2]. Good health is not only the absence of disease or infirmity; a person-centred, subjective perspective offers an understanding of the quality, not just the quantity, of survival. HRQoL is used in health research, for example, used to assess the clinical and cost-effectiveness of interventions [3]. In clinical practice, HRQoL measurement can enable clinicians and patients to understand the impact of a health condition and treatment on a patient’s life and enhance clinical decision-making [4].

The measurement of HRQoL in children has received insufficient attention. The UK’s NICE 2013 methods guide [5] recommends a preferred HRQoL measure for adult trials but states there is insufficient evidence to recommend a measure for children. More research is needed in this area [3, 6, 7]. Designing HRQoL measures for children is complex because of their developmental characteristics [4, 6], including the complexity around the extent to which children can reliably report on their health and the extent to which proxy-reports are appropriate alternatives. To our knowledge, no research has explored professional’s perspectives on the challenges of paediatric HRQoL measures and new approaches to advance the field.

Digital ecological momentary assessment (EMA) is a data collection method characterised by brief, repeated sampling of an individual’s thoughts, emotions, experiences and behaviours, in-the-moment and in-the-context [8], using technology (e.g. smartphones and smartwatches) to capture user-reported data as individuals go about their everyday lives. Digital EMA may be better suited to child self-report. In comparison with traditional HRQoL measures, which can be lengthy and reliant on retrospective reporting, the brevity of EMA may minimise burden on the child, and in-the-moment administration may minimise recall bias [9, 10].

EMA has been used with young people, with high completion rates, high satisfaction rates, feasibility and reliability [11, 12]. A systematic review of mobile-technology-based EMA with youth included five studies with young participants (7 and 8 years), with the compliance rate ranging from 59–92% [9]. There is an evidence gap around the use of EMA with younger children (under 7 years). The same technologies used for digital EMA can also capture passive/sensing data, providing information about behaviours, physiology, and context (e.g. via accelerometery, electrocardiogram and geolocation-tracking [13]). This data could be a proxy for HRQoL where child and proxy reporting may be unreliable or impossible (e.g. due to very young age or physical disability) and may also provide insights into discrepancies between child and parent/carer HRQoL reports [14]. To our knowledge, Digital EMA and passive/sensing data approaches have not been used for paediatric HRQoL.

We aimed to explore professionals' views (clinicians and researchers) to identify the challenges of measuring HRQoL in children (5–11 years) and whether digital EMA and passive/sensing data could enhance HRQoL measurement.

## Methods

### Study design, recruitment and sampling

This was a qualitative study involving one-to-one semi-structured interviews. We recruited clinicians and researchers with experience using HRQoL measures with children (5–11 years) via professional networks and snowballing [15]. We sent invitations via the National Institute for Health and Care Research (NIHR) paediatric methodology incubator, the Centre for Development, Evaluation, Complexity and Implementation in Public Health Improvement (DECIPHer), and the Centre for Academic Child Health (CACH, University of Bristol). The recruitment channels were UK-based, and so we anticipated only recruiting experts from within the UK. We also used snowballing, and because of this, we ended up recruiting three participants from outside of the UK (see Table 2). Study invitations included a participant information sheet and an online expression of interest (EOI) form (captured via REDCap [16]). The EOI form captured contact details, sampling and eligibility data (demographic and professional characteristics). We purposively sampled, aiming for diversity in the professional background, geography, gender, and ethnicity of participants. We contacted sampled participants and obtain consent via REDCap. Participants were offered a £20 voucher for participation. We undertook iterative sampling, analysis and sampling until reaching data saturation [17]. We ended recruitment when we achieved a diverse sample (particularly considering professional backgrounds), when we were no longer creating additional codes, and when we determined additional interviews were no longer adding additional information (saturation).

### Data collection

We conducted one-off interviews designed to last 60 min, conducted face-to-face or online via Microsoft Teams. Only the interviewer and participant were present. The semi-structured topic mapped onto our research questions: the challenges of measuring HRQoL in children (section one), views on using Digital EMA and passive/sensing data to capture HRQoL (section two), see Table 1. Interviews were audio recorded, transcribed verbatim using a professional transcription service, and pseudonymised.

**Table 1:** Overview of topic guide

Section	Overview
One	Having prior experience with paediatric HRQoL measures was an inclusion criterion for the study. We began with an open-ended exploration of participants' views of the challenges of paediatric HRQoL measurement, encouraging participants to draw upon their concrete experiences with these measures (“Tell me about your experiences using HRQoL measures with children (5–11 years”). We chose to start with this section as we believed this would create a scaffold from which we could move to more hypothetical reflections on how digital EMA and sensing data might address the challenges of paediatric HRQoL measurement. Within section 1, we included prompts to encourage participants to reflect on how the challenges of HRQoL capture may vary with age, across childhood (5–11 years). This was based on the existing literature about child development being a fundamental issue for paediatric HRQoL measurement [4], and with different approaches typically being taken for younger children (5–7 years) and older children (8–11 years)
Two	Prior knowledge or experience with digital EMA and sensing health data was not an inclusion criterion. To address the potential lack of knowledge/ experience, we provided participants with a two-minute video outlining the main characteristics of Digital EMA. We then asked open questions to explore participants' views on the application of these methods/technologies to paediatric HRQoL capture. As this was the first study (to our knowledge) to explore Digital EMA and sensing data in relation to paediatric HRQoL, we designed the questions in this section to be open and exploratory (“Can you talk about Digital EMA as an approach for HRQoL measurement?”), with follow-up prompts to explore views on potential benefits and problems of the approach. We chose not to base questions around a theory or framework (such as the Unified Theory of Technology and Acceptance [18]), so as not to constrain participants in their thinking about the potential challenges and opportunities. We encouraged participants to reflect on the challenges of HRQoL measures that they had raised in section one as a basis for their reflections on the application of EMA and sensing data to HRQoL

### Analysis

We analysed data thematically [19] (AB, HF, LT), with each author analysing the transcript from their completed interview. Following initial familiarisation with the data, we completed independent coding of the of the first set of transcripts undertaking line-by-line coding. We then meet to review the initial codes and collate these into a coding framework, which encapsulated all the initial codes. We set up the coding framework in NVivo [20] and applied this to transcripts. The coding framework was refined as additional transcripts were analysed. Through repeated discussion, we refined codes and grouped these into themes and sub-themes. Analysis was inductive and driven by the data.

### The research team

Interviews and analysis were conducted by LT (a female PhD student, using qualitative methods in her PhD and with a Health Psychology MSc), HF (a female PhD student with a background in interdisciplinary psychology and neuroscience, and with previous experience of qualitative methods), AB (a female Lecturer, who used qualitative methods during her MSc and PhD). The authors (AB, MR, EC) had experience in paediatric clinical trials using HRQoL measures, and this experience stimulated their interest in improving paediatric HRQoL measures. The researchers were interested in EMA applied to paediatric HRQoL, but had no conflicts of interest in this line of research. For most interviews, there was no existing relationship between the participant and interviewer. In two cases, the interviewer had previously worked in the same University Faculty but had a limited prior relationship.

## Results

### Participants

We received 26 EOI forms. We interviewed 18 participants, see Tables 2 and 3.

**Table 2:** Summary of demographic characteristics

Professional and demographic characteristics		Frequency (%)
Gender	Female	11 (61%)
	Male	5 (30%)
	Preferred not to say	2 (11%)
Geographical region	Southwest England	10 (56%)
	Not from the UK	3 (17%)
	Northwest England	2 (11%)
	Northeast England & Cumbria	1 (6%)
	West Midlands	1 (6%)
	Yorkshire and the Humber	1 (6%)
Ethnicity	White	16 (89%)
	Preferred not to say	2 (11%)
Researcher/clinician*		14/8

*Some participants identified as having both a clinical and research professional background

**Table 3:** Themes, subthemes and illustrative quotes

Theme	Sub theme	Illustrative quotes
1: The challenges of child-reported HRQoL	1.1 Conceptualisation and domains	*What even is quality of life*? (ID 6, Epidemiologist)
	1.2 Reflecting on and reporting HRQoL	*it’s that recall–they can’t necessarily remember what happened two months ago, and you haven’t seen them for three months and* [you don’t have information on when they] *were low, and they potentially didn’t go to school and they potentially didn’t play, that makes a huge impact on how quickly you step up their treatment.* (ID 8, Dermatologist)
	1.3 Individualising HRQoL measurement	*Maybe you shouldn’t have necessarily minimum age or maximum age for these scales, because sometimes it depends on the child, and their maturity and their experience* (ID 5, Research Project Manager)
2: The challenges of proxy-reported HRQoL	2.1 Can proxy observers meaningfully report on the child’s subjective experiences?	[of proxy reporting] *How much confidence would we place that this really, truly reflects a child’s health status really?* (ID 16, health economist)
	2.2 Discrepancies between the child and proxy	*The child may have a different opinion to the parent* (ID 8, Dermatologist)
3: Making sense of changes in HRQoL over time		*How do you have measures that also adapt to things like growth and maturity. It's really a challenge.* (ID 5, Research Project Manager)
4: Digital EMA as a solution?	4.1 EMA and the trade-off between richness of data and burden	*Being able to track things longitudinally and being able to look back on that would be a really helpful clinical tool.* (ID 6, Epidemiologist) *it’s really cool that you can collect that* [sensing data] *so like to supplement … I think it’s like not about having exactly the same thing captured, for instance talking about sleep … I can see that you slept for eight hours last night but how was your quality of sleep*. (ID 1, Clinical Trial Statistician) *you’ll end up having these really difficult choices about jettisoning things that you know they would be really nice to know but they’re just not going to be important enough* (ID 7, general practitioner/academic)
	4.2 EMA for a child-centred approach?	*Very tailored to the child* (ID 8, Dermatologist)
	4.3 Practical and ethical concerns	*Wearables… issues about them being in school and not being allowed to wear them. We’ve also had some discussions with kids about, wearing them to sleep and parents not wanting them to wear them to sleep, or them finding uncomfortable* (ID 6, epidemiologist)

### Themes

**Fig. 1:**
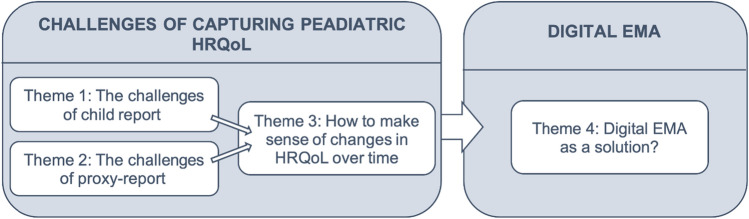
Overview of themes

We developed four themes. See Fig. 1 for an overview of themes; Table 3 for themes, subthemes and illustrative quotes; and the supplementary file for additional supporting quotes.

## Theme one: The challenges of child-reported HRQoL

### Conceptualisation and domains

Participants talked about HRQoL being a complex construct, which is difficult to conceptualise for children and changes with maturation. Participants highlighted that there is one “*main measure*” (Participant 4, health economist) for adults (the EQ-5D). In contrast, for children, there is not one established measure, reflecting uncertainty in conceptualisation.

Participants questioned whether the domains included in measures were “*applicable*” for all children. Some participants questioned the extent to which children can “*unpick*” (Participant 5, research project manager) different domains of HRQoL- for example, questioning children’s ability to distinguish mood from physical discomfort. Some participants suggested dropping discrete domains favouring a single, global assessment. Whilst others felt it was important to keep multi-dimensional domains to maintain sensitivity and to “*capture the heterogeneity in kids’ quality of life”* (Participant 6, Epidemiologist).

Participants questioned the extent to which children have the capacity to engage in preference tasks to determine utility weighting from domains. This value preference work has yet to be done in children under eight years, and participants said it was unclear whether the older children (around eight years) did engage appropriately with these judgement tasks.

### Reflecting on and reporting HRQoL

Participants talked about children reflecting on their HRQoL in a more immediate way, being less likely to reflect back over long time periods and being more influenced by the immediate context. Clinicians described the challenge of obtaining a complete, accurate clinical picture when the child’s account is restricted to recent events and the impact of this on determining appropriate treatment.

Being influenced by the immediate context was described as problematic in circumstances where the child is required to complete measures outside of their day-to-day environments (e.g. in clinic), leading to responses “*not representative of how they might feel day to day living their life.”* (Participant 5, research project manager), i.e. not ecologically valid.

Participants also talked about children being more “*sensitive*” (Participant 3, paediatrician/researcher) in how they experience or report their HRQoL, resulting in higher variability. This can create problems when measuring HRQoL infrequently, with infrequent measurements failing to capture fluctuation and creating “*guesswork*” between the measured time points.

Another proposed difference in how children reflect on their HRQoL is that they are quicker to adapt or habituate to illness, with one participant stating that children become “*blind to how burdensome [the] current situation has become*” (Participant 11, nurse/research fellow). As such, over time, a child may be more likely to report improvement in HRQoL in the continued presence of objective illness/disability.

Because of these differences in how children report HRQoL, some participants said it might not be valid to ask younger children about their HRQoL (“[HRQoL measures] *can’t be used in under-eights”,* participant 6, epidemiologist). Others said children should not be underestimated, saying it is important to give children the “*benefit of the doubt*” (Participant 11, nurse/research fellow).

### Individualising HRQoL measurement

Themes 1.1 and 1.2 highlight the difference in how children conceptualise, reflect on and report their HRQoL. These differences are tied to development, and as children mature, so too will their conceptualisation, reflection and reporting of HRQoL. Participants highlighted that HRQoL measures typically deal with these developmental changes by using fixed age bounds for measures. Participants described this fixed approach as incongruous with the highly individual nature of child development. As an alternative, they suggested selecting measures based on the child’s individual ability rather than age.

To respond to individual differences, participants suggested tailoring domains and tailoring weightings to the individual.

Participants also talked about individualising the formats of measures, offering formats tailored to the child's preferences (e.g. text versus visual). Further, participants described a need to tailor measures to different clinical contexts, for example, acute versus chronic conditions.

## Theme two: The challenges of proxy-reported HRQoL

### Can proxy observers meaningfully report on the child’s subjective experiences?

Participants questioned whether proxy observers could accurately report on the child's internal experiences and to what extent this is “*guessing*” (Participant 6, epidemiologist), inaccurate or biased. This was seen as particularly problematic for domains such as well-being and pain. Participants suggested parent’s may be unable to disentangle their own QoL from their child’s, with their own well-being colouring their perceptions of their child’s health. Further, participants raised the issue that some parents may be motivated to misreport their child’s HRQoL.

Because of these challenges, one participant wondered whether it was more appropriate for the proxy to report on their own HRQoL rather than the child’s, with the parent’s own HRQoL relevant because "*how a child is feeling or their quality of life is intrinsically linked to the parent*." (Participant 14, health economist).

### Discrepancies between the child and proxy

Participants flagged potential "*discrepancy”* between parent and child reports, driven by different conceptualisations, priorities, and perspectives; child reports and proxy reports are “*not really the same data point”* (Participant 5, research project manager). For example, parents may take a longer-term view of the child’s HRQoL compared to the child’s immediate perspective (as described in theme 1.2). Participants had different views on whether the child or proxy report is more accurate, and one participant questioned the notion of one view being more “a*ccurate*” than the other. In general participants said that as children get older, there is a reduced use of parent-proxy and an increased privileging of the child’s perspective.

## Theme three: Making sense of changes in HRQoL over time

Theme one highlighted the child’s changing conceptualisation and reporting of HRQoL with maturation. Theme two described the decreasing need for parent-proxy reports as the child matures. These changes over time create a challenge for “*consistency*” (Participant 4, health economist) and continuity of HRQoL measurement and a challenge for understanding changes in HRQoL over time. Does a change in HRQoL over time reflect a real change in health status, or does it reflect a change in how children are conceiving/ reporting their HRQoL or a transition from proxy-report to child report?

## Theme four: Digital EMA as a solution?

In this section, we use “EMA” to refer to self and proxy-reported data and “passive data” to refer to any non-actively reported data (e.g. sensing data).

### EMA and the trade-off between richness of data and burden

Theme one described the challenges of infrequent data collection and variability of HRQoL in children. Many participants talked about the value of EMA repeated measurement in this context. Researchers spoke about the “*really rich data set*” (Participant 18, academic) produced by repeated measures, the increased ability to capture variability, the “*peaks and troughs*” (Participant 10, health economist) and “*get a better picture of what’s going on”* (Participant 10, health economist). Clinicians discussed the value of more data points in between appointments, with in-the-context data enabling an understanding of “*triggers*” (Participant 9, nurse consultant) and the impact of “*setting*” on the child’s experiences (Participant 9, nurse consultant). One participant described repeated measures between appointments as a mechanism for remote monitoring and reducing the need for clinic visits.

However, some participants were concerned that increased self/proxy reporting would increase burden on respondents, with children unlikely to have sustained engagement and likely to get “*bored*” (Participant 6, epidemiologist), particularly for younger children (under eights). There were concerns that this burden could cause bias, with the characteristics of those likely to engage with a demanding protocol, different to those who do not. Another point raised was the impact of repeatedly probing into sensitive topics, such as mental health, and the burden of lack of improvements being made salient to participants.

Many participants were positive about the richness of data that could result from collecting passive data from wearables alongside self-report EMA. They highlighted the value of this rich data being collected in a “*non-burdensome”* (Participant 11, nurse/research fellow) manner. Participants talked about using GPS, sleep, HR and accelerometery data as objective markers alongside self/proxy-reported HRQoL. Participants felt objective markers should be used to supplement self/proxy-report data rather than replace it, suggesting objective markers could add another dimension of understanding to the child’s health state, a quantitative measure to complement the quality of experiences.

Some participants flagged the potential for discrepancy between the objective (passive data) and subjective (EMA) data. Some raised this as a challenge, wondering how these discrepancies would be managed. Others felt that discrepancies would raise “*interesting questions about how well perceived health corresponds with the [objective] measurement*.” (Participant 16, health economist). One participant described the clinical benefits, saying that an objective sense of severity would be helpful in addition to the child/parent report, which may be distorted.

Participants also reflected on the burden placed on clinicians and researchers using and analysing the rich, complex datasets produced by repeated EMA and passive data. Researchers would need to consider resourcing specialist statistical training or data analysis support, and there would need to be consideration of how the complex data could be analysed in line with established Health Economic analysis guidelines (e.g. how to develop quality adjusted life years from HRQoL measures). Participants flagged the significant groundwork needed to make sense of the passive/sensing data; understanding norms, confounders, and highly variable data. Clinicians recognised the lack of time/capacity to work with a volume of raw longitudinal EMA and passive data. They expressed a need for succinct insights and overviews, through effective data visualisation.

*Reconciling richness and burden* Participants discussed methodological solutions to address the tension of richness versus burden arising from EMA. Item reduction could mitigate the impact of the burden of repeated measurement. However, there were divergent views about item reduction. Some stated briefer measures (even “*one question”*) would be “*more likely to gain acceptance*”, "*more valuable in clinical practice*" (Participant 13, general practitioner) and are typically used for health economic evaluation. Others were concerned about losing valuable, “*multifaceted*” (Participant 10, health economist) information. Participants did raise the need for short measures to be “*validated*” and “*reliable*” (Participant 15, researcher). Another methodological solution was the use of “*short, discrete*” (Participant 2, paediatrician/academic) periods of EMA measurement to offset the burden (similar to the concept of measurement bursts [21]).

When making decisions around balancing richness and burden of EMA, weighing up methodological choices like frequency, item reduction and measurement bursts, participants said that the decision-making needed to be tailored. Tailoring to the specific health context of use (e.g. acute conditions needing more frequent measurement to understand changes than chronic), the individual needs of the child, considering factors such as age, and whether it was being used in a clinical versus research context. In some contexts, the priority would be capturing multifaceted data (longer measures over fewer time points); in other contexts, the priority would be temporal richness (repeated measures with briefer measures).

### EMA for a child-centred approach?

*Individualisation* Participants described digital EMA as having the potential to offer a variety of formats for viewing and responding to measures, e.g. audio, images, text and even features such as “*gamification*” (Participant 4, health economist). The ability to offer different formats, adapted/tailored to the individual’s needs and preferences, could increase accessibility and make the measures more meaningful to the child. For example, it could facilitate offering “*simple imagery*” for younger children.

*Appropriate for the child’s stage of cognitive development* Theme one described the more immediate way children reflect on their HRQoL and the effects of this on recall and ecological validity. Many participants said EMA properties of in-the-moment and in-the-context measurement might address these challenges.

### Practical and ethical concerns

Participants raised the point that "*primary school children aren’t likely to have phones*" (Participant 14, health economist). Some talked about wearable devices (e.g. smartwatches) as a practical and appropriate technologies for this age group, though some still felt this was inappropriate for younger children (under eights). Participants raised concerns about the practicality of administering HRQoL measures via wearable devices, emphasising that solutions needed to be “*workable and simple enough”* (Participant 7, general practitioner/academic). There were concerns about the child’s ability to navigate the technology independently, particularly for younger children (those under eight years). There were also the risks of devices being lost, forgotten or left uncharged; the inability to wear the devices in certain contexts (e.g. whilst at school, during physical exercise lessons, or whilst asleep); and discomfort or aggravation caused by devices. These were also ethical concerns about inequality and inclusion for groups of children without access to new technologies or where their health condition would be a barrier to use (e.g. children with eczema having skin aggravation), as well as concerns about the social impact of the child wearing a visible device (e.g. stigma and bullying) or EMA prompts being too “*intrusive*”.

There were also concerns about the practical impact on clinicians, for example, the time and cost of integrating new technologies with existing systems, purchasing licenses and training staff. Ethical concerns included the added “*responsibility and clinical governance”* (Participant 13, general practitioner) of managing sensitive digital health data.

### Accuracy and functionality of technology

In general, a digital approach was seen as “*preferable to a paper-based*" (Participant 10, health economist), likely to “*reduce the barriers*” (*Participant 5, research project manager*), and improve completion rates. Participants described the benefits of wearable technology; the measure being physically present (“*on your arm*”), the ability to use digital “*reminders*” (Participant 10, health economist) to prompt EMA completion and the reduced likelihood of losing questionnaires. In terms of smartphone administration, participants commented on the ubiquity of smartphones and preferences for phone-based rather than paper-based measures.

However, participants did flag the limitations of the accuracy of sensing technologies. Further, they highlighted the “*limited functionality*” (Participant 8, dermatologist) of wearable devices for children due to the need for increased controls to ensure the security of children’s devices).

## Discussion

### Summary of findings

This is the first study to explore clinicians' and researchers' views about the role of Digital EMA and passive data in addressing the challenges of measuring paediatric HRQoL. Key challenges included: uncertainty around conceptualising paediatric HRQoL, with conceptualisation likely to change with maturity; the distinct way children report HRQoL (more immediate, sensitive, and variable); recall issues, compounded by infrequent measurement; and significant individual differences across childhood, incongruous with the fixed content and formats of HRQoL measures. Digital EMA may address these challenges. In-the-moment and in-the-context measurements may address recall issues and ecological validity. Repeated measurement may help with issues of variability, whilst also providing richer datasets. Technology could enable personalisation of content (domains/items) and formats. A key consideration of using Digital EMA is the trade-off between richness and burden, with this trade-off being different for different contexts. There were concerns over the methodological, practical, and ethical challenges of Digital EMA and passive data collection. There were also challenges identified about paediatric HRQoL measurement, which Digital EMA would not clearly address. For example, the challenge of interpreting changes in HRQoL over time (does a change in HRQoL over time reflect a real change in health status, or is it due to developmental changes).

*Individualisation/tailoring* Our findings highlight the challenge of using fixed/static HRQoL measures with a diverse population, in terms of the child’s development and health context. Technology could be used to tailor HRQoL measures to the context and individual, with the tailoring of protocols (e.g. frequency, item reduction, measurement bursts [21]), content (domains and items), and formats (e.g. text vs visual). Existing technologies enable the individualisation of outcome measurement. Mesmerise software [22] enables researchers to set-up tailored EMA protocols (e.g. tailoring content, frequency and timings of prompts), as well as enabling respondents to set-up individualised parameters for their EMA schedule (e.g. specifying time limits for receiving prompts). There are examples of health-tracking Smartphone Applications designed to administer a core of fixed domains, with users able to select additional tailored domains from a pre-set list of options [23]. Computerised Adaptive Testing [24] uses responses to generate the next question, reducing the number of questions needed to provide an accurate T-score. Technology has also been used to offer a range of formats of outcome measures, including interactive visualise designs [25], video [26] and voice assistants. We are not aware of the use of these novel technologies for paediatric HRQoL, and this warrants further research.

*Objective markers* Our findings highlight that the subjective nature of HRQoL is one of the fundamental challenges of measuring HRQoL in children: can children reliably report HRQoL? Can proxies meaningfully report on the subjective experiences of children? These findings are consistent with existing literature, for example, findings of discrepancies between child and proxy reports of the child’s HRQoL [27, 28]. Participants described using objective passive/sensing data from wearables as objective markers to complement data on the quality of experience. Using objective markers aligns with Lin et al.’s conceptualisation that “HRQoL includes both subjective and objective components… objective assessment focuses on what the individual can do, such as walking” [2]. Our participants described the additional benefit of objective data placing minimal burden on respondents, consistent with findings that wearable activity monitors foster greater tracking adherence than manual tracking [29]. Future research could explore digital objective markers as a compliment to HRQoL measurement, with attention to issues around accuracy of sensing data, the complexity of managing the outputs (clinically and in research), and practical and ethical concerns about data collection via wearables.

*Continuity of measurement, in the context of development changes* Participants hypothesised that reflections/reporting of HRQoL changes with development. Younger children may adapt to illness quicker and be more immediate and sensitive in their reflections/reporting of HRQoL (leading to higher variability). This hypothesis, that the mean and variability of HRQoL change with development, is a hypothesis that HRQoL measures are statistically non-stationary [30]. This poses the question- are changes in HRQoL over time due to a change in health status or due to developmental changes in how children report HRQoL? This is worthy of further exploration.

### Strengths and limitations

We recruited participants from a range of professional backgrounds, from across England. As such, it is likely that we have captured a range of practices and perspectives on HRQoL measurement. Our robust analysis procedures ensured we represented the range of views, including divergent/conflicting opinions. We reached data saturation [17] for our main aims.

Participants typically provided rich reflections on using HRQoL measures, drawing on their experiences. In contrast, some participants were unfamiliar with EMA, so although all participants did offer their thoughts on EMA, for many these were reflections on a hypothetical concept. Reflections based on real-life technology usage can be more beneficial than hypotheticals [31], and piloting with user feedback would be a beneficial next step for this research. Further limitations include an all-female group of researchers undertaking interviews and analysis, as well as a lack of ethnic diversity in the participant group. However, we think it is unlikely that gender or ethnicity would significantly influence research findings around paediatric HRQoL measurement in the UK. Finally, transcripts were professionally transcribed to ensure data quality. However, transcripts were not returned to participants for comment, which could have enhanced data quality. Further, participants were not asked to provide feedback on the findings.

## Conclusion

Digital EMA may address some of the issues of paediatric HRQoL measurement. Potential avenues for future research include investigating the use of technology to individualise and tailor HRQoL measures for children. Equally, the role of passive data to supplement HRQoL could be explored, with attention to accuracy and interpretation, the conceptual issues around combining subjective and objective data and the ethical and practical aspects of using wearable devices with children.

### Supplementary Information

Below is the link to the electronic supplementary material.

Supplementary file1 (PDF 200 kb)
