# Supplementary material for: "not one size fits all” The challenges of measuring paediatric health-related quality of life and the potential role of digital ecological momentary assessment: a qualitative study
Source: Qual Life Res. 2023 Oct 27;33(2):443–52. doi: 10.1007/s11136-023-03535-6 (PMC10850023; doi:10.1007/s11136-023-03535-6)
Supplement: Supplementary file 1 — Supplementary file1 (PDF 200 kb) [file 11136_2023_3535_MOESM1_ESM.pdf]

## Supplementary file: Table of themes, subthemes and illustrative quotes.

| Theme | Sub Theme | Illustrative quotes |
|---|---|---|
| ***1: The challenges of child-reported HRQoL*** | 1.1 Conceptualisation and domains | *What even is quality of life*? (ID 6, Epidemiologist) |
| | 1.2 Reflecting on and reporting HRQoL | *thrown off by the little perturbations* (ID 3, Paediatrician and Researcher)<br><br>*it's that recall – they can't necessarily remember what happened two months ago, and you haven't seen them for three months and* [you don't have information on when they] *were low, and they potentially didn't go to school and they potentially didn't play, that makes a huge impact on how quickly you step up their treatment.* (ID 8, Dermatologist)<br><br>*my main gripe … is the fluctuating health states … we can only ask questionnaires at several timepoints throughout a 12-month trial and is that really representative of quality of life*. (ID 10, Health Economist) |
| | 1.3 Individualising HRQoL measurement | *not one size fits all* (ID 9, Nurse Consultant)<br><br>*all children develop at different stages... there's so much variability it's quite tricky* (ID 8, Dermatologist)<br><br>*Maybe you shouldn't have necessarily minimum age or maximum age for these scales, because sometimes it depends on the child, and their maturity and their experience* (ID 5, Research Project Manager)<br><br>*how much weight should you put on the physical versus the mental... Does sleep matter more to you than pain?' … should there even be a different weighting for each child – would that be more helpful?* (ID 6, Epidemiologist)<br><br>*many of the tools have not particularly well adapted for those very short-term changes*. (ID 7, General Practitioner/Academic) |
| ***2: The challenges of proxy-reported HRQoL*** | 2.1 Can proxy observers meaningfully report on the child's subjective experiences? | *parents kind of guessing at their child's quality of life* (ID 6, Epidemiologist)<br><br>[of proxy reporting] *how much confidence would we place that this really, truly reflects a child's health status really?* (ID 16, health economist)<br><br>*it's like if you're having a really bad day ... you've just applied for some benefits to help with your child's disability … and you've found out that you haven't received them. In the same day, your clinician sends you a form - how do you think your child's disease is impacting their life today? You're probably gonna have a different response to a day where they're doing something positive.* (ID 5, research project manager)<br><br>*Anxiety, depression type things possibly. If a parent wants to underplay, downplay.* (ID 14, health economist) |

| | | |
|---|---|---|
| | | *we don't know whether we're measuring predominately the child's quality of life or the parent's quality of life or the parent's perception of the child's quality of life… sometimes I think we could be a little bit more brave and just say let's just measure the parent's quality of life and use that as a proxy* (ID 7, general practitioner/academic) |
| | 2.2 Discrepancies between the child and proxy | *the child may have a different opinion to the parent* (ID 8, Dermatologist) |
| ***3: Making sense of changes in HRQoL over time*** | | *how do you have measures that also adapt to things like growth and maturity. It's really a challenge.* (ID 5, Research Project Manager)<br><br>*its challenging for when the young people start reporting it themselves …and then we had to try and compare, okay, has the score changed because their quality of life has changed or has it changed because it's gone from parents recording it to the children recording it?* (ID 6, Epidemiologist) |
| ***4: Digital EMA as a solution?*** | 4.1 EMA and the trade-off between richness of data and burden | *drowning in data* (ID 7, General Practitioner and Academic)<br><br>*being able to track things longitudinally and being able to look back on that would be a really helpful clinical tool.* (ID 6, Epidemiologist)<br><br>*If the people's health fluctuates a lot I can see that there will be huge advantages in using the method [EMA].* (ID 16, Health Economist)<br><br>*if children don't need to come to hospital for tests, then they shouldn't come to hospital for tests. If they can monitor themselves at home or they can use you know wearable technology that monitors … then it should be encouraged.* (ID 5, Research Project Manager)<br><br>*respondent burden… when we ask a lot of people we'll get a small proportion who do everything for us... we can lean an enormous amount from them but … they do generally tend to be the less socio economically deprived and better educated people.* (ID 7, General Practitioner/Academic)<br><br>*I can imagine getting more frustrated if you knew.. If the results weren't changing* (ID 10, Health Economist)<br><br>*I am not worried, sad or unhappy…you could ask several times a day, but I suppose I'd worry a little bit with that one about whether it might make them think too much about being worried, sad or unhappy.* (ID 14, Health Economist)<br><br>*it's really cool that you can collect that* [sensing data] *so like to supplement … I think it's like not about having exactly the same thing captured, for instance talking about sleep … I can see that you slept for eight hours last night but how was your quality of sleep*. (ID 1, Clinical Trial Statistician) |

| | | |
|---|---|---|
| | | *I don't know what you would do if it was discrepant or you know the kids like I slept for six hours and you were like actually no according to the wearable you slept for eight hours*. (ID 1, Clinical Trial Statistician)<br><br>*if you've got GPS then you know for example a child has gone to school* (ID 7, General Practitioner/Academic)<br><br>*With EMA, I'm kind of struggling to understand how you would even analyse it because you've got so, so much data and I think it's I think that's going to be a real challenge to analyse it to get something out of it…* (ID 1, Clinical Trial Statistician)<br><br>*its got to be easy to navigate and easy to see … a quick pictorial representation of how that child's been I think is what you're looking for to aid the clinician without taking up too much time because everybody is under time constraints* (ID 8, Dermatologist)<br><br>Reconciling richness and burden<br><br>*you'll end up having these really difficult choices about jettisoning things that you know they would be really nice to know but they're just not going to be important enough* (ID 7, General Practitioner/Academic) |
| | 4.2<br>EMA for a child-centred approach? | Individualisation<br><br>*very tailored to the child* (ID 8, Dermatologist)<br><br>Appropriate for the child's stage of cognitive development<br><br>*it's always a problem isn't it if people are having to recall things that happened a while ago or even last week or whatever, if they are doing it with something that's really fresh in their memory that's got to be beneficial.* (ID 18, Academic)<br><br>*if you could provide technology that means they complete that form at home where they're more comfortable, it's probably more accurate in that respect.* (ID 5, Research Project Manager) |
| | 4.3<br>Practical and ethical concerns | Really *actually delivering it* (ID 3, Paediatrician and Researcher)<br><br>*Wearables… issues about them being in school and not being allowed to wear them. We've also had some discussions with kids about, wearing them to sleep and parents not wanting them to wear them to sleep, or them finding uncomfortable* (ID 6, epidemiologist)<br><br>*We got anecdotal feedback... people who said if this was electronic I would complete it, but paper, I am disinclined to complete this paper-based questionnaire … most people have smart phones, people are on their phones* (ID 18, academic) |
